# Male Breast Cancer: Treatment Trends, Reported Outcomes, and Suggested Recommendations

**DOI:** 10.7759/cureus.18337

**Published:** 2021-09-27

**Authors:** Evelina Arzanova, Harvey N Mayrovitz

**Affiliations:** 1 Osteopathic Medicine, Nova Southeastern University Dr. Kiran C. Patel College of Osteopathic Medicine, Davie, USA; 2 Medical Education, Nova Southeastern University Dr. Kiran C. Patel College of Allopathic Medicine, Davie, USA

**Keywords:** treatment recommendations, treatment outcomes, surgical procedures, mastectomy, breast conservative therapy, sentinel lymph node biopsy, male breast cancer

## Abstract

Male breast cancer (MBC) is unfamiliar to most men, and its optimal treatment options are not well recognized by many treating physicians. The lack of MBC specific clinical trials contributes to the limitations of understanding MBC specific pathology, treatment options, and outcomes. This state-of-affairs contribute to perpetuating the use of treatment methods derived from our existing knowledge of female breast cancer (FBC). Previous studies demonstrate that men are often undertreated or disproportionately treated using more invasive surgical procedures even in the early stages of MBC. The purpose of this investigation was to thoroughly discuss current MBC treatment options, provide an evidence-based summary of their outcomes, note recent improvements, discuss important considerations and recommendations. Our goal is to aid the treatment decision process for patients and treating physicians.

## Introduction and background

A study from as early as 1978 reported that men were diagnosed with male breast cancer (MBC) around the median age of 65 years and most presented in later stages of their disease (stage II, 54%) with infiltrating ductal carcinomas (87%) [1]. Regardless of their age or stage at diagnosis, those with­­­­­ axillary lymph node involvement had lower ten-year survival rates (11%) than women of similar age and axillary lymph node invasion (43%) [1]. Four decades later, men are still diagnosed at a median age of 63.3 years [2]. They present in later stages (stage III 14%, stage IV 5.8%) of breast cancer than women (Stage III 8.9%, stage IV 3.85) and more often with estrogen receptor-positive (ER+) (83.9%) ductal cell carcinomas (75.5%) [2]. They now have a 14.6% lower survival rate than women with breast cancer [2].

MBC is only 1% of all annually diagnosed breast cancers in the United States [3]. Nevertheless, its age-adjusted incidence across all races and ethnicities increased by 40% from 1975 to 2015, nearly doubling the 24.7% incidence increase in FBC [4]. The majority of those affected by MBC are black men, who have a 52% higher incidence rate than white men [5]. Overall, the increase in incidence is still on the rise, and it is estimated that in 2021 approximately 2,650 men will be diagnosed with breast cancer, while 530 of them will die from this disease [6].

Several risk factors have been associated with an increased chance of developing MBC, such as older age (≥70 years), family history of breast cancer in the first-degree relative, and hereditary genetic mutations (*BRCA2* more commonly than *BRCA1*) [7,8]. Other risk factors include (a) Klinefelter syndrome, a congenital genetic abnormality resulting in a 47XXY genotype causing small testicles, lower levels of androgen hormones, and increased estrogen hormones, (b) use of exogenous estrogen, previously used as a treatment for prostate cancer, (c) obesity, and (d) history of chest radiation [8].

A painless breast mass is a common presenting symptom of breast cancer [9,10]. If the mass is deemed suspicious for cancer after a diagnostic imaging test such as mammograms, a breast tissue biopsy can be obtained to definitively establish cancer diagnosis most of the time [10]. In addition, an axillary (underarm) lymph node biopsy can be used to determine its anatomical extent [10]. Cancer cells can be described, graded (I-III), and staged based on their type (e.g. ductal or lobular), apparent degree of differentiation, and using the 8th edition of the American Joint Committee on Cancer (AJCC) tumor, nodes, and metastases (TNM) classification system of malignant tumors, respectively [10]. In the TNM staging system, the T stands for the primary tumor size (0-4), the N denotes lymph node spread (0-3), and M indicates metastasis (0-1) [10]. Biological markers such as hormone receptor presence (ER+/-, PR +/-) and Her2+/- protein production are also assigned [10]. Once the cancer stage is determined, treatment options such as surgery, chemotherapy, hormone treatment, radiation, and other therapies can be considered [10]. 

Surgery is the most common treatment, as indicated by multiple studies in which over 90% of MBC patients (n >10,000) were reported to be surgically treated [11,12]. MBC patients, when treated by surgery, had an increase in postoperative survival (surgery hazard ratio (HR)=0.49, p< 0.001) as compared with alternative treatments [12]. However, every surgical procedure presents its own set of risks and possible acute and chronic complications, which are not well elaborated in the literature on MBC [13]. Based on available data collected from 2007-16, 4.6% of men reported postsurgical complications within 30 days following any surgical breast cancer management, with 3.2% of those being wound complications (superficial surgical site infection (SSI) 2.4%, deep SSI 0.5%, organ space SSI 0.2%, wound disruption 0.1%) [14]. As a result, less invasive surgical treatment approaches have been implemented in breast cancer treatment [14,15]. Yet, men are still treated with mastectomy 86% of the time, while 50% of women have a less invasive breast conservative surgery (BCS), suggesting that men with MBC are not always treated in accordance with the FBC treatment guidelines [16].

The lack of MBC specific clinical trials [17], screening guidelines [7], and trial-based standard of care treatment options [18], along with limited reports on treatment-associated complications [13], makes choosing the optimal treatment difficult for patients and treating physicians [19]. Surveys show that 36% of recently diagnosed men wanted more male-specific breast cancer information on surgical treatment options, sentinel node biopsies, prophylactic mastectomies, and non-surgical treatments [19]. Similarly, 79% of treating healthcare professionals searched for specific MBC information, indicating the need for the present evidence-based summary and analysis [19].

## Review

The purpose of this review was to analyze recent literature on the management of MBC and provide an evidence-based summary of the current surgical treatments while noting any changes, advances, complications, survival outcome statistics, or recommendations. The goal is to aid the treatment decision process for MBC patients and treating physicians. 

Sentinel lymph node biopsy and axillary lymph node dissection

A sentinel lymph node is the primary lymph node that directly drains the breast cancer area [10]. Once identified, the node is biopsied to determine if any cancer cells are present [10]. This process is referred to as a sentinel lymph node biopsy (SLNB). If cancer cells are detected, a more extensive surgical procedure called axillary lymph node dissection (ALND) is usually done in which multiple lymph nodes are removed [10]. 

Treatment Trends

ALND was the more common, if not only, axillary treatment for decades until SLNB was first used in MBC patients in the mid to late 1990s [20]. This change was associated with fewer complications than ALND, such as a decreased incidence of lymphedema [20]. Study reports show a 27% increase in the use of SLNB from 2006-16 [21]. A more recent study of SLNB use reported that in a group of 354 MBC patients, 53% had an SLNB [22]. This was an increase as compared to earlier studies. Overall, the use of SLNB is slowly increasing, but ALND is still the more common procedure used in men [23]. 

Treatment Outcomes

Multiple study findings report that SLNB has a high success rate with minimal postsurgical comorbidities and breast cancer recurrence [17,24]. For instance, in a study of 25 MBC patients with no significant difference in age, tumor location, and axillary node involvement, sixteen men (64%) had an SLNB, and nine men (36%) had an ALND [17]. Based on the results of both procedures, four men (25%) from the SLNB group and three (33%) from the ALND group had pathologically node-negative breast cancers (pN0, not detected based on histological lymph node analysis) [17]. Four years later, men who had an SLNB did not report any sensorimotor upper limb concerns or breast cancer recurrence; however, in those who had an ALND, eight men (89%) complained of sensory or motor arm deficits on the surgical side, and one patient reported axillary cancer recurrence [17]. The authors concluded that SLNB was used successfully in all intended patients and correlated with fewer postsurgical comorbidities [17]. Contributors also believe that if SLNB was the initial treatment of choice based on the clinical absence of node involvement (clinically node-negative (cN0), not detect during a physical exam), 33% of men who had an ALND could have avoided the associated post-ALND complications [17]

Similarly, another study in 32 MBC patients reported a 100% identification rate of sentinel lymph nodes and no breast or axillary cancer recurrence on follow-up 30 months later [24]. Based on the results of this study, authors believe that SLNB should be the initial treatment of choice in MBC patients with clinically negative lymph nodes [24]. The American Society of Clinical Oncology (ASCO) also states that SLNB leads to fewer complications than an ALND including, the postoperative development of lymphedema, shoulder discomfort, sensory deficits, infections, and arm-related morbidity [15]. 

Recommendations

The consensus of the ASCO is that SLNB should be equally accurate in men and women [15]. Thus, it should be presented as a choice for qualified men using the same criteria as used in women. The criteria are: 70 years or younger, with clinically negative nodes, and having no significant comorbidities [15]. Additionally, the Society of Surgical Oncology (SSO) does not recommend routine use of SLNB in patients who are 70 years or older and have cN0, HER2+ cancer, and being treated with hormone therapy [25]. SSO recommendations state that there is no increase in cancer mortality or local recurrence associated with not doing a SLNB based on multiple studies [25,26].

Breast-conserving surgery and mastectomies

Breast-conserving surgery (BCS), commonly referred to as a lumpectomy, involves the elimination of cancer cells along with a cancer-free margin to guard against possible residual cancer cells left behind [27,28]. Consistent with FBC treatment guidelines, men treated by BCS, regardless of nodal involvement, should receive postsurgical radiation therapy (RT) to remove any remaining cancer cells and lower the risk of local cancer recurrence [29]. When BCS is combined with RT, it is referred to as breast-conserving therapy (BCT) [29]. Alternatively, mastectomies are more invasive surgical procedures that involve eliminating breast tissue (ducts, lobules (if present), and fatty connective tissue) or more depending on the degree of cancer spread [27].

Treatment Trends 

According to the reviewed studies, BCS is underused in MBC patients, with most patients treated by mastectomies[11,23,30].Of additional concern is the absence of follow-up radiation treatments seen in patients treated by BCS [11]. For instance, of 10,873 MBC cases with stage I (37.9%) and stage II (43.4%) ductal carcinomas, most were treated with a total mastectomy (71.3%) rather than BCS (24%) [11]. Further, only 70% of those who chose BCT received postsurgical RT [11]. This was also reported for 1,054 men with non-metastatic breast cancer, in which only 4.1% of men were treated with BCS, and 45% of them did not receive the recommended adjuvant RT [23]. Of the majority who had a mastectomy (95.9%), 30.7% had cancer-positive lymph nodes [23]. Despite this, they did not receive RT [23].

In general, the purpose of BCS is to provide a survival rate equivalent to that of a mastectomy, a cosmetically acceptable breast, and a low rate of cancer recurrence in the treated breast [31]. However, study authors suggest that BCS is often used as a form of palliative care and not for its intended benefits [30]. According to their study, BCS was the treatment of choice in older patients (≥ 80 years) with stage IV or unknown stages of breast cancer, who did not undergo a lymph node biopsy compared to the younger early-stage breast cancer patients [30]. 

The reported predominance of mastectomy use [11,23,30] could be due to several factors. These factors include: patient avoidance of RT if their cancer is confined to the breast tissue only, tumor position within the central and retro-areolar location making BCT impossible [9], habitual use of old practice patterns [23], or lack of adherence to the suggested breast treatment guidelines [30]. Another reason could be the lower male preference placed on preserving the appearance of the breast, although study results show that 44% (four out of nine) MBC patients requested to have BCT or a mastectomy with breast reconstructive surgery [32]. An additional 4.2% of 1773 men asked for immediate breast reconstruction, demonstrating that some men do desire to preserve their breasts' natural look [14].

Treatment Outcomes

Research shows that survival rates of MBC patients treated by BCS and RT are near equal if not better than patients treated by mastectomy [30,33,34]. In a study of 1777 MBC cases, where 17% of men had BCS and 40% had a radical or simple mastectomy, the five-year case-specific survival rate was equal amongst the treatment groups only if all mastectomy patients had stage I cancer and postsurgical radiation therapy [34]. However, the need for RT did not significantly impact the survival of patients treated by BCS, of whom only 46% received it [34]. Additionally, BCS treatment made no statistical difference in the overall survival of 752 men 70 years or older with stage I, ER+ cancer, treated with or without post-surgical RT compared to mastectomy [33]. Similarly, a 0.5% ten-year survival and 5.5% greater overall survival (OS) were reported in men ages 80 or older with stage IV MBC if treated by BCS rather than mastectomy [30]. The results reported by these studies suggest that using less invasive procedures is safe even in men of greater age and severity of cancer [30,33]. 

The consistent survival rates attributed to BCT are further supported by the retrospective analysis of 8445 cases of MBC patients with early-nonmetastatic cancer [28]. Patients who had a mastectomy alone (61.2%) or mastectomy with RT (12.4%) had a lower OS compared to the 18.2% who had BCS with radiation (p<0.001 all) [28]. The ten-year survival rate was 73.8% for BCT with RT, 58.0% total mastectomy alone, 56.3% for partial mastectomy alone, and 56.3% for a total mastectomy with RT [28].

Important Treatment Considerations 

Several studies have reported that men treated with BCS have low postoperative compliance with radiotherapy and hormone therapy treatments, thus not completing the entire BCT (i.e., surgery and post-operative treatment) [35,36]. The percentage of radiotherapy non-compliance reported ranged between 27-46% in the analysis of seven retrospective studies, with the exception of one study that reported a compliance rate of 86% [36]. While another study found that treatment with tamoxifen, a hormone therapy drug that blocks estrogen receptors in ER+ cancers after BCS to help prevent cancer reoccurrence [37], was discontinued in 20.3% of 64 MBC patients at a median time of 49-months [35]. Reasons for discontinuation most often included weight gain (22%) and sexual dysfunction (22%) [35]. 

Recommendations 

It has been 31 years since the National Institutes of Health (NIH) announced that the less invasive BCS followed by RT is an appropriate procedure with an equivalent survival rate to mastectomy used in treating early FBC [38]. Based on the FBC guideline recommendations and multiple study findings showing equal survival rates in those treated by BCS and mastectomy [11,28,30,33], qualified men should consider BCS along with postsurgical RT as a safe treatment option. Additionally, the authors suggest treating physicians should note the lack of postoperative therapy compliance and assist patients in making a well-informed decision when selecting their cancer treatment [36]. 

Nipple and skin-sparing mastectomies

The nipple-areolar complex (NAC), sparing mastectomy, preserves the dermis and epidermis of the nipple while removing the ducts from within the lumen [39]. 

*Treatment Trends and Outcomes* 

Literature on the use of NAC sparing mastectomies in men is sparse, possibly due to an insufficient number of performed procedures [40]. During 2007-16, only 1.1% of 1773 men had a NAC sparing mastectomy [14]. However, case study reports suggest that NAC sparing mastectomies are feasible in men [39,40]. Based on a case study report by Lanitis et al., a centrally located, 1cm, grade II invasive ductal carcinoma with one positive lymph node was successfully removed, sparing the nipple and areola [40]. This patient had no recurrence of cancer eight years later [40]. A second case study by Noor et al. was based on an incidental finding of a bilateral ductal carcinoma in-situ during surgery for apparent gynecomastia. The patient was treated with a nipple and skin-sparing mastectomy bilaterally with no cancer recurrence reported at his 18-month treatment follow-up [39].

Important Treatment Considerations 

Choosing the NAC sparing mastectomy as treatment should be done with the understanding that there is limited research concerning cancer recurrence and outcomes of this type of mastectomy in men [39]. Additionally, early and late complications associated with NAC sparing mastectomies, as seen in women, should be further reviewed to aid in the treatment decision process [41]. 

Recommendations 

Mastectomies sparing the nipple and skin often provide the best cosmetic result, thus attracting those individuals who wish to undergo reconstructive breast surgery if the tumor has not affected the NAC significantly [42]. Decisions regarding how much skin to preserve, whether the NAC can be spared or removed, and the appropriate incisions to use depends mainly on the initial tumor's size and surgeons’ recommendations on a case-by-case basis [42].

Mastectomies: simple, modified radical, and radical

The three most used mastectomies in men are: (a) total mastectomy that is whole breast amputation, (b) modified radical mastectomy or whole breast amputation with level I and II axillary node clearance (>10 lymph nodes), and (c) radical mastectomy or whole breast amputation including all axillary lymph nodes and pectoralis major [27].

Treatment Trends 

Prior to 1970, radical mastectomies were the gold standard for MBC treatment, according to a review of 229 cases during 1955-66 [43]. However, less invasive procedures such as modified radical mastectomies (MRM) became the primary surgical treatment, based on the analysis of 106 cases during 1975-90 [9]. This study reported that 67% of men had a MRM compared to the 26% of men treated with a total radical mastectomy. However, the most performed surgical treatment during 2007-16 was a simple mastectomy in 46.7% of 1773 men [14]. The second most common surgical treatment was MRM (34.2%), and the least common was radical mastectomy (2%) [14]. Based on these trends, it is apparent that although mastectomies are still more common in men than BCT [11-12], amongst mastectomy procedures, less invasive mastectomies are rising in popularity [9, 14].

Treatment Outcomes

With the introduction of BCT, research has focused on comparing BCT versus mastectomy outcomes. However, one study of 50 MBC cases found no difference in survival outcomes (p=0.8) between 45 men who had a radical or modified mastectomy and five men who had a simple mastectomy [44]. 

Important Treatment Considerations 

Although survival outcomes are similar between mastectomies and BCT [11, 28, 30, 33], postoperative complications should be considered. These complications include infections, seromas with fluid collection surrounding the wound that can become a nexus for infection, and lymphedema [45]. Study results show that men have a three-time greater risk of developing postoperative seromas than women [46]. Non-wound-related complications can also arise [14]. Based on the analysis of 1773 MBC cases treated surgically, 1.7% had non-wound-related complications such as pneumonia, bleeding requiring blood transfusions, and deep vein thrombosis, as well as unplanned hospital readmissions [14].

Recommendations

Despite similar survival outcomes amongst mastectomies and BCT [11,28,30,33], not every type of cancer qualifies for BCT, and mastectomies might be recommended for some MBC patients. These include those who have advanced stage III breast cancer, large invasive tumors (T2 >5cm) even after neoadjuvant hormone therapy, Paget’s disease of the breast (a rare type of cancer involving the nipple skin and areola), recurrent breast cancer in an area previously treated with RT, or inflammatory breast cancer [45]. 

Prophylactic mastectomy

Prophylactic mastectomy is the removal of the contralateral breast in a patient diagnosed with breast cancer or someone at a higher risk of developing breast cancer (*BRCA2* mutation carrier, recurrent breast cancer), in which case both breasts are removed [27]. 

Treatment Trends 

Previous studies have consistently demonstrated an increase in the number of individuals treated with bilateral mastectomies [11, 14, 47]. Between 2004-5 and 2010-11, the rates of contralateral prophylactic mastectomies (CPM) in men increased by 86.7% [47]. Based on literature findings, the number of CPM in MBC patients has been increasing in the following fashion: 2004-11, 4.4% of (n=6332), 2004-14, 6.1% of men (n=10327) [11] and 2007-16, 6.7% (n=1773) [14].

*Important Treatment Considerations* 

Strong evidence-based data suggests that although bilateral mastectomies might prevent breast cancer reoccurrence, they do not improve overall survival [47,48]. 

Recommendations 

In a survey of 1,226 therapeutic and radiation oncologists and plastic surgeons, 92% recommended CPM for patients with a mutated *BRCA* gene, and 60.2% of those surveyed discourage CPM for patients with only an average risk [49]. The presence of a *BRCA* gene mutation correlates with the most considerable support for getting a CPM [49]. For patients that do not fall into the high-risk category, a new nomogram is available that is designed to predict the risk of developing cancer in the contralateral breast and may help guide the choice for getting a CPM [50]. This nomogram was developed based on medical information collected from 4,405 MBC patients treated with a unilateral mastectomy or CPM from 1998 to 2015 and can estimate the three-, five-, and eight-year breast cancer-specific death in MBC survivors [50]. Overall, the benefits of CPM are best weighed against its risks for those considering contralateral or bilateral prophylactic mastectomies [47]. 

## Conclusions

Male breast cancer is a rare and understudied disease with an increasing annual incidence and need of future research. The present review has confirmed a steady increase in the use of some but not all less invasive surgical procedures in the management of MBC. Reports on the use of SLNB show a slowly increasing trend with successful lymph node identification rates and few reported post-procedural complications, such as lymphedema and limb paresthesia. However, there is also evidence of apparent underuse of BCS, hormone, and radiation therapies. Without male-specific treatment trials, we can only reason that the predominant retro-areolar tumor growth of MBC and tumor invasion secondary to late-stage presentation of this disease might account for the low use of BCS. While poor patient compliance and the absence of concrete male-specific treatment guidelines might be contributing to the inconsistent use of non-surgical treatment options. A decrease in the use of radical mastectomies compared to simple mastectomies, in favor of less invasive treatments, has also been observed. 

Overall, based on a synthesis and integration of the findings present in this investigation, we believe the following recommendations appear warranted:

(a) The BCT option should be discussed, along with the likely need for postsurgical RT and long-term hormone treatments. Precisely, greater emphasis should be placed on discussing the long-term potential for side effects and patient compliance with the use of hormonal and radiation therapy. 

(b) Mastectomies are best used for patients who require them based on discussions of benefits, risks, and long-term complications. 

(c) There is a need for MBC specific clinical trials to better classify the histological, genetic, and pathological uniqueness of this disease and to establish male-specific treatment guidelines not necessarily based on existing evidence of FBC. 

(d) Identifying men at risk for MBC, utilizing the screening guidelines for high-risk patients, and raising awareness of this disease should be done to increase early-stage cancer diagnosis and chances of survival.
